# Differential expression of the BCAT isoforms between breast cancer subtypes

**DOI:** 10.1007/s12282-020-01197-7

**Published:** 2020-12-24

**Authors:** Mai Ahmed Shafei, Arwa Flemban, Carl Daly, Paul Kendrick, Paul White, Sarah Dean, David Qualtrough, Myra E. Conway

**Affiliations:** 1 Faculty of Health and Life Sciences, University of the West of England, Coldharbour Lane, Bristol, BS16 1QY UK; 2 Department of Pathology, Faculty of Medicine, Umm Al-Qura University, Makkah, 24382 Saudi Arabia

**Keywords:** BCAT, IDH, HER2 +, Luminal A, Breast cancer

## Abstract

**Background:**

Biological characterisation of breast cancer subtypes is essential as it informs treatment regimens especially as different subtypes have distinct locoregional patterns. This is related to metabolic phenotype, where altered cellular metabolism is a fundamental adaptation of cancer cells during rapid proliferation. In this context, the metabolism of the essential branched-chain amino acids (BCAAs), catalysed by the human branched-chain aminotransferase proteins (hBCAT), offers multiple benefits for tumour growth. Upregulation of the cytosolic isoform of hBCAT (hBCATc), regulated by c-Myc, has been demonstrated to increase cell migration, tumour aggressiveness and proliferation in gliomas, ovarian and colorectal cancer but the importance of the mitochondrial isoform, hBCATm has not been fully investigated.

**Methods:**

Using immunohistochemistry, the expression profile of metabolic proteins (hBCAT, IDH) was assessed between breast cancer subtypes, HER2 + , luminal A, luminal B and TNBC. Correlations between the percentage and the intensity of protein expression/co-expression with clinical parameters, such as hormone receptor status, tumour stage, lymph-node metastasis and survival, were determined.

**Results:**

We show that hBCATc expression was found to be significantly associated with the more aggressive HER2 + and luminal B subtypes, whilst hBCATm and IDH1 associated with luminal A subtype. This was concomitant with better prognosis indicating a differential metabolic reliance between these two subtypes, in which enhanced expression of IDH1 may replenish the α-ketoglutarate pool in cells with increased hBCATm expression.

**Conclusion:**

The cytosolic isoform of BCAT is associated with tumours that express HER2 receptors, whereas the mitochondrial isoform is highly expressed in tumours that are ER + , indicating that the BCAT proteins are regulated through different signalling pathways, which may lead to the identification of novel targets for therapeutic applications targeting dysregulated cancer metabolism.

## Introduction

Breast cancer is the most common malignancy in women accounting for almost one in four cancer cases and is responsible for approximately 627,000 deaths each year globally [1]. It is a heterogeneous disease with several distinctive molecular subtypes which are defined by the status of oestrogen receptor (ER), progesterone receptor (PR) and overexpression/amplification of human epidermal growth factor receptor 2 (HER2) [2]. Molecular profiling has led to the identification of four intrinsic molecular subtypes; luminal A, luminal B, HER2 + and triple-negative breast cancer (TNBC; ER-/PR-/HER2-) which represent biologically distinct disease entities [3, 4]. Luminal A breast cancers are characterized by high expression of luminal epithelial genes and low expression of Ki-67 [3], unlike Luminal B breast cancers, which have higher Ki-67 expression. Luminal B sub-type also has lower expression of several luminal-related genes (such as *ESR1* or *FOXA1*), genomic instability and a higher frequency of *TP53* gene mutations associated with a worse prognosis and a higher risk of relapse than luminal A breast cancers [4–6]. Both luminal A and luminal B are characterised by the expression of the hormone receptors ER and PR, whilst a proportion of luminal B tumours are *HER2*-enriched [2]. HER2 + breast cancer is characterised by *HER2* amplification and the low expression of luminal and basal clusters [2, 3] whilst TNBC is characterised by tumours that lack the expression of ER and PR, the absence of *HER2* overexpression, and high expression of Ki-67 [2].

Prognosis and treatment strategies vary greatly between breast cancer molecular subtypes due to the activation of different oncogenic pathways [7]. A significant improvement in breast cancer survival rates through the introduction of therapies that target hormone receptors, such as Tamoxifen (targets ER + tumours) and Trastuzumab (targets the HER2 tumours), has been reported [8, 9]. Despite the advancement in HER2-targeted therapy, HER2 continues to be one of the most aggressive subtypes of breast cancer with high mortality rates [10]. Moreover, breast cancer recurrence is a common contributor to breast cancer death rates either by metastasis or chemo-resistance [11, 12]. There is, therefore, an unmet need for the identification of novel therapeutic targets downstream of these growth receptors, which may improve the therapeutic efficiency.

Altered cellular metabolism is a fundamental adaptation of cancer during disease development [13]. Many metabolic pathways have been reported to be dysregulated in breast cancer leading to metabolite addiction, such as dependency on glutamine for tumour growth, which can be exploited in cancer therapy [14, 15]. This reprogramming of cell metabolism in breast cancer is initiated by activation of oncogenes, such as *c-Myc*, which plays a central role in orchestrating proliferation, metabolism and differentiation. A target of c-Myc is the human cytosolic branched-chain aminotransferase protein (hBCATc). The hBCAT proteins exist in two main isoforms, the mitochondrial hBCAT isoform (hBCATm protein, *BCAT2* gene) is widely expressed in most tissues, whereas the cytosolic hBCAT (hBCATc protein, *BCAT1* gene) is restricted to highly specialised tissues including brain and placenta [16]. Increased levels of the BCAT gene, *BCAT1* have been extensively reported in malignancies including gliomas [17, 18], ovarian [19], colorectal [20], gastric cancer [21], nasopharyngeal carcinomas [22], breast cancer [23] and chronic myeloid leukemia (CML) [24]. In gliomas, increased hBCATc and hBCATm expression has been limited to gliomas with wild-type isocitrate dehydrogenase 1 (*IDH1*), cytosolic and *IDH2*, mitochondrial [17, 18]. As a result, perturbations in BCAA metabolism have also been a subject of increased interest [25] where upregulation of hBCATc has been reported to increase cell proliferation and migration in ER-negative breast cancer cells [23]. Our recent study showed that hBCATc regulates TNBC cell proliferation migration and invasion through the IGF-1/insulin PI3K/Akt pathway, culminating in the upregulation of FOXO3a and Nrf2, pointing to a novel therapeutic target for breast cancer treatment [26].

The IDH enzymes which catalyse the oxidative decarboxylation of isocitrate to produce the TCA intermediate α-ketoglutarate, also support cell growth [27]. *IDH* mutations have been found to result in neomorphic activity to metabolize α-ketoglutarate to R-2-hydroxyglutarate (2HG), an oncometabolite, which accumulates and inhibits chromatin-modifying enzymes and has been demonstrated to inhibit hBCATc expression in gliomas [28–31]. In contrast to gliomas, there is little evidence of *IDH1* mutations in breast cancer, with only one reported case noted [32, 33]. The association between IDH and hBCAT expression has not been evaluated in breast cancer, investigation of which may uncover novel associated pathways. We hypothesise that these metabolic pathways converge in tumour cells, where particular metabolic pathways are favoured differentially between breast cancer subtypes.

In this study, hBCATm was found to be significantly associated with IDH1 expression, indicating that these two metabolic pathways are activated concomitantly. Expression of hBCATm and IDH1 correlated with luminal A breast cancer and smaller breast tumours, indicating better prognosis. Differentially, hBCATc expression was found to be significantly associated with the more aggressive HER2 + and luminal B subtypes identifying subtype-dependent metabolic liabilities. Understanding the metabolic profiles of the different subtypes of breast cancer could provide novel tailored therapeutic targets.

## Materials and methods

### Materials

Rabbit-raised monoclonal antibody to IDH1 was purchased from Abcam (Cambridge, UK). Rabbit-raised monoclonal antibody to hBCATc and hBCATm was purchased from Insight Biotechnology Limited (Wembley, UK). ImmPACT DAB Peroxidase (HRP) Substrate and Vectastain elite ABC kit were purchased from Vector Labs (Peterborough, UK). DPX-mountant, Harris’s Haematoxylin, were purchased from Sigma-Aldrich (Dorset, UK). All other chemicals were purchased from Thermo Fisher Scientific (Loughborough, UK).

### Breast cancer tissue samples

Archival human breast cancer tissue samples from surgical resections of breast tumours were obtained from the Bristol Royal Infirmary under ethical approval from NHS Health Research Authority and University of the West of England, Ethics Committee (Ref. 11/SW/0127). To re-confirm phenotype, all cases were IHC stained for ER, PR, HER2 and Ki67. Luminal A breast cancer was classified as tumours with ER/PR-positive staining, low Ki67 (< 20%) levels and negative for HER2 overexpression, whilst luminal B breast cancer was defined as ER/PR-positive, HER2-negative/positive with high Ki67 (20%). HER2 breast cancer subtype was defined in tumours with negative ER/PR and HER2 overexpression. Breast tumours which lacked expression of ER/PR and overexpression of HER2 were defined as TNBC. The patient cohort consisted of a total of 83 breast cancer cases which were classified into subtypes: HER2 + subtype (18 cases), luminal A (19 cases), luminal B (10 cases) and TNBC (36 cases). Patients’ clinical pathological characteristics included hormone receptor status, tumour stage, lymph-node metastasis and survival (Table 1).

**Table 1:** Clinicopathological characteristics of the patient cohort according to breast cancer subtypes

Parameters	Total (*n* = 83) (%)	TNBC (*n* = 36) (%)	Luminal A (*n* = 19) (%)	Luminal B (*n* = 10) (%)	HER2 (*n* = 18) (%)	*p* value
Age (year, mean ± SD)	61.04 ± 13.1	62.85 ± 13.0	61.06 ± 14.0	55.59 ± 16.3	60.44 ± 10.0	0.6799
ER						**< 0.001**
Negative	52 (64.2)	35 (100.0)	1 (5.3)	1 (10.0)	15 (88.2)	
Positive	29 (35.8)	0 (0.0)	18 (94.7)	9 (90.0)	2 (11.8)	
PR						**< 0.001**
Negative	58 (71.6)	35 (100.0)	5 (26.3)	3 (30.0)	15 (88.2)	
Positive	23 (28.4)	0 (0.0)	14 (73.7)	7 (70.0)	2 (11.8)	
HER2						**< 0.001**
Negative	57 (70.4)	35 (100.0)	15 (78.9)	5 (50.0)	2 (11.8)	
Positive	24 (29.6)	0 (0.0)	4 (21.0)	5 (50.0)	15 (88.2)	
Tumour type						0.159
Lobular	9 (10.8)	6 (16.7)	3 (15.8)	0 (0.0)	0 (0.0)	
Ductal	68 (81.9)	27 (75.0)	15 (78.9)	8 (80.0)	18 (100)	
Mixed/other	6 (7.2)	3 (8.3)	1 (5.3)	2 (20.0)	0 (0.0)	
Histological grade						**< 0.001**
I/II	42 (50.6)	11 (30.6)	18 (94.7)	6 (60.0)	7 (38.9)	
III	41 (49.4)	25 (69.4)	1 (5.3)	4 (40.0)	11 (61.1)	
Tumour stage						0.208
T1	43 (52.4)	17 (48.6)	14 (73.7)	4 (40.0)	8 (44.4)	
T2	34 (41.5)	16 (45.7)	3 (15.8)	5 (50.0)	10 (55.6)	
T3	5 (6.1)	2 (5.7)	2 (10.5)	1 (10.0)	0 (0.0)	
Nodal stage						0.074
N0	54 (65.9)	25 (71.4)	15 (78.9)	5 (50.0)	9 (50.0)	
N1	18 (22.0)	6 (17.1)	2 (10.5)	3 (30.0)	7 (38.9)	
N2	7 (8.5)	3 (8.6)	2 (10.5)	0 (0.0)	2 (11.1)	
N3	3 (3.7)	1 (2.9)	0 (0.0)	2 (20.0)	0 (0.0)	**0.03**
TNM stage						
I	35 (42.7)	15 (42.9)	13 (68.4)	4 (40.0)	3 (16.7)	
II	35 (42.7)	15 (42.9)	4 (21.1)	3 (30.0)	13 (72.2)	
III	12 (14.6)	5 (14.3)	2 (10.5)	3 (30.0)	2 (11.1)	
Tumour size (cm)						0.162
≤ 2	46 (57.5)	18 (52.9)	13 (68.4)	8 (80.0)	7 (41.2)	
> 2	34 (42.5)	16 (47.1)	6 (31.6)	2 (20.0)	10 (58.8)	

Significant *p* values are indicated in bold

Kruskal–Wallis test was used to assess significant differences for age and linear by linear association used for statistical analysis of all other parameters

### Immunohistochemical staining and scoring

Formalin-fixed, paraffin-embedded (FFPE) tissues were serially sectioned at 4 µm using a microtome (Leica RM2235) and mounted on Superfrost Plus slides (Thermo Fisher Scientific, Loughborough, UK). Tissue sections were deparaffinised in histoclear (National Diagnostics, Atlanta, GA, USA) and rehydrated using a series of ethanol concentrations and dH_2_O. Endogenous peroxidase was quenched in 3% v/v hydrogen peroxide for 10 min at room temperature. Antigen retrieval was performed by heating sections in 10 mM citrate buffer pH 6.0 boiled at 95 °C for 30 min using a water bath and then allowing the sections to cool to room temperature in the buffer. Non-specific binding sites were blocked with 5% horse serum in Tris-buffered saline (TBS) (200 mM sodium chloride, 2 mM tris, pH 7.5) for 1 h at room temperature and sections were incubated with primary antibody (1:200 for hBCATc, 1:800 hBCATm and 1:400 IDH1) in blocking serum overnight at 4 °C. Rabbit polyclonal antibody raised to hBCATc and rabbit polyclonal antibody hBCATm were obtained from Insight Biotechnology Limited (Wembley, UK) and rabbit polyclonal antibody raised to IDH1 was obtained from Abcam (Cambridge, UK). Sections were washed twice with TBS and incubated with biotinylated antibody to IgG in TBS for 1 h followed by avidin–biotin complex in TBS incubation for 30 min (Vectastain ABC kit; Vector Laboratories, Peterborough, UK). Slides were subsequently developed with 3,3′-diaminobenzidine (ImmPACT DAB Peroxidase (HRP) Substrate DAB; Vector Laboratories, Peterborough, UK) for 10 min. Sections were counter-stained with Haematoxylin (25% w/v Harris’s Haematoxylin). Slides were then dehydrated with graded ethanol, cleared in histoclear and mounted using DPX (Sigma-Aldrich, Dorset, UK). Secondary antibody and only controls were included. The sections were examined on a light microscope and scored using the IRS scoring system as described in Table 2. Independent scoring was performed by two immunohistochemists to validate the score assigned. Representative images were captured on a Nikon Eclipse 80i (Nikon UK, Kingston Upon Thames, UK).

**Table 2:** IRS and nuclear expression scoring system

Percentage of positive cells	Intensity of staining	IRS class (0–12)
0 = no positive cells	0 = no reaction	0–1 = negative
1 ≤ 10% of positive cells	1 = mild reaction	2–3 = mild
2 = 10–50% positive cells	2 = moderate reaction	4–8 = moderate
3 = 51–80% positive cells	3 = intense reaction	9–12 = strongly positive
4 ≥ 80% positive cells		

IRS—points	IRS classification (0–3)
0–1	0 = negative
2–3	1 = positive, weak expression
4–8	2 = positive, moderate expression
9–12	3 = positive, strong expression

Nuclear - score	Nuclear expression (0-3)
0	No nuclear staining
1	< 10% of tumour cells have nuclear staining
2	10-50% of tumour cells have nuclear staining
3	≥ 51% of tumour cells have nuclear staining

### Statistical analysis

Immunohistochemical data were analysed using SPSS (Version 23; SPSS Inc., Chicago, IL, USA) for Mac. Statistical significance was reported when *p* < 0.05. Chi-square was used to analyse associations between breast cancer subtypes and clinicopathological characteristics. Linear-by-linear association was used to investigate the relationship between the expression of the metabolic proteins and clinicopathological characteristics. The Kruskal–Wallis test was used to assess age differences between breast cancer subtypes. Kaplan–Meier survival curves and log-rank statistics were used to evaluate time to tumour recurrence and overall survival.

## Results

**Fig. 1:**
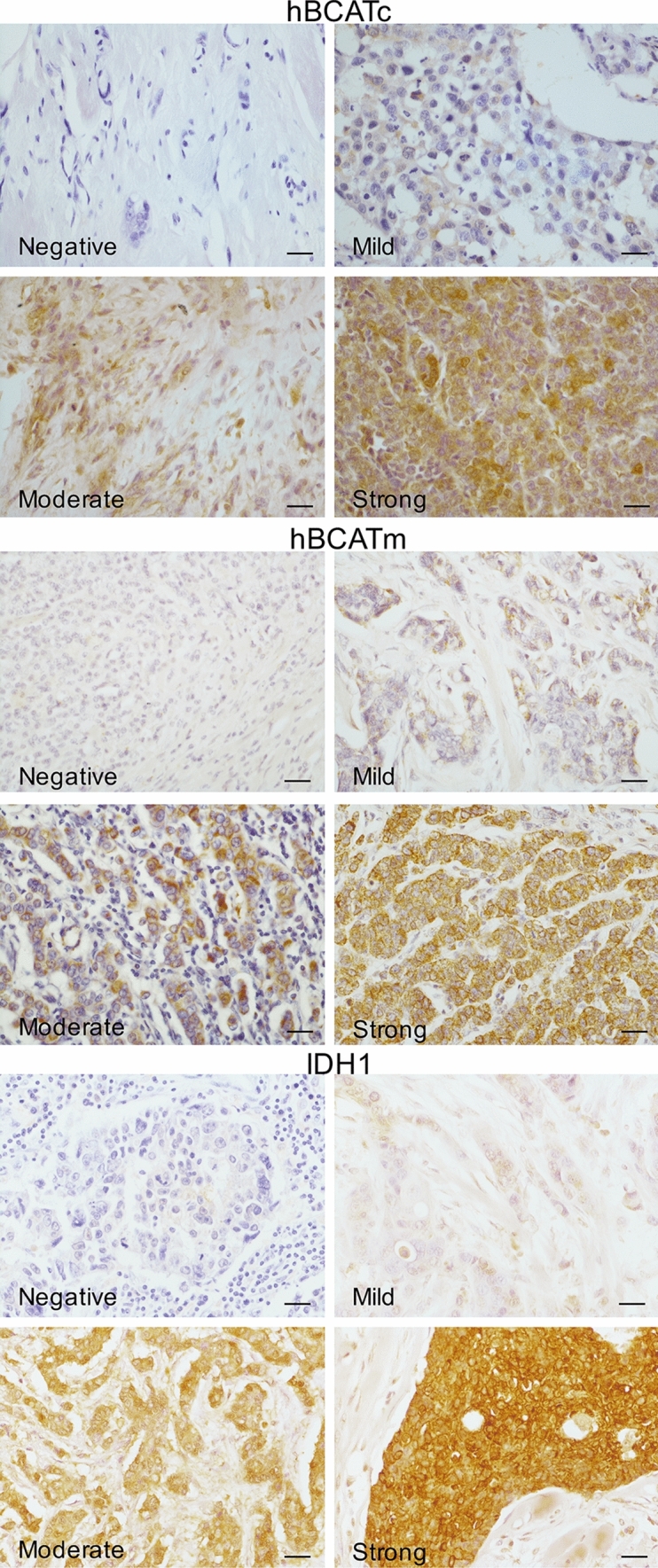
Representative images of the intensity of immune reaction for each of the metabolic proteins, as indicated, in human breast cancer sections. Slides were scored using the IRS scoring system and images captured by Leica microscope using a × 40 objective (scale bars = 25 μm)

Scoring of protein expression of the various metabolic proteins in the human breast cancer tissue was conducted using the IRS scoring system as described in Table 2. To allow comparability, the intensity scores were adjusted with a score of 4 allocated to the highest intensity and 1 for the lowest positive signal, to account for variation of antibody and protein expression for each of the metabolic proteins as illustrated in Fig. 1. More than 75% of cases showed positive expression for the metabolic proteins tested (Table 3). Analysis using the IRS scoring system allowed associations between the percentage and the intensity of protein expression with clinical parameters and co-expression of the markers to be elucidated in this study.

**Table 3:** Table presenting the number of positive and negative breast cancer sections for each of the metabolic markers from total cases assessed

	Negative (%)	Positive (%)	Total
hBCATc	28 (37.3)	47 (62.7)	75
hBCATm	9 (11.7)	68 (88.3)	77
IDH1	7 (9.2)	69 (90.8)	76

### hBCATc expression associated with HER2 + breast cancer subtype and receptor status

**Fig. 2:**
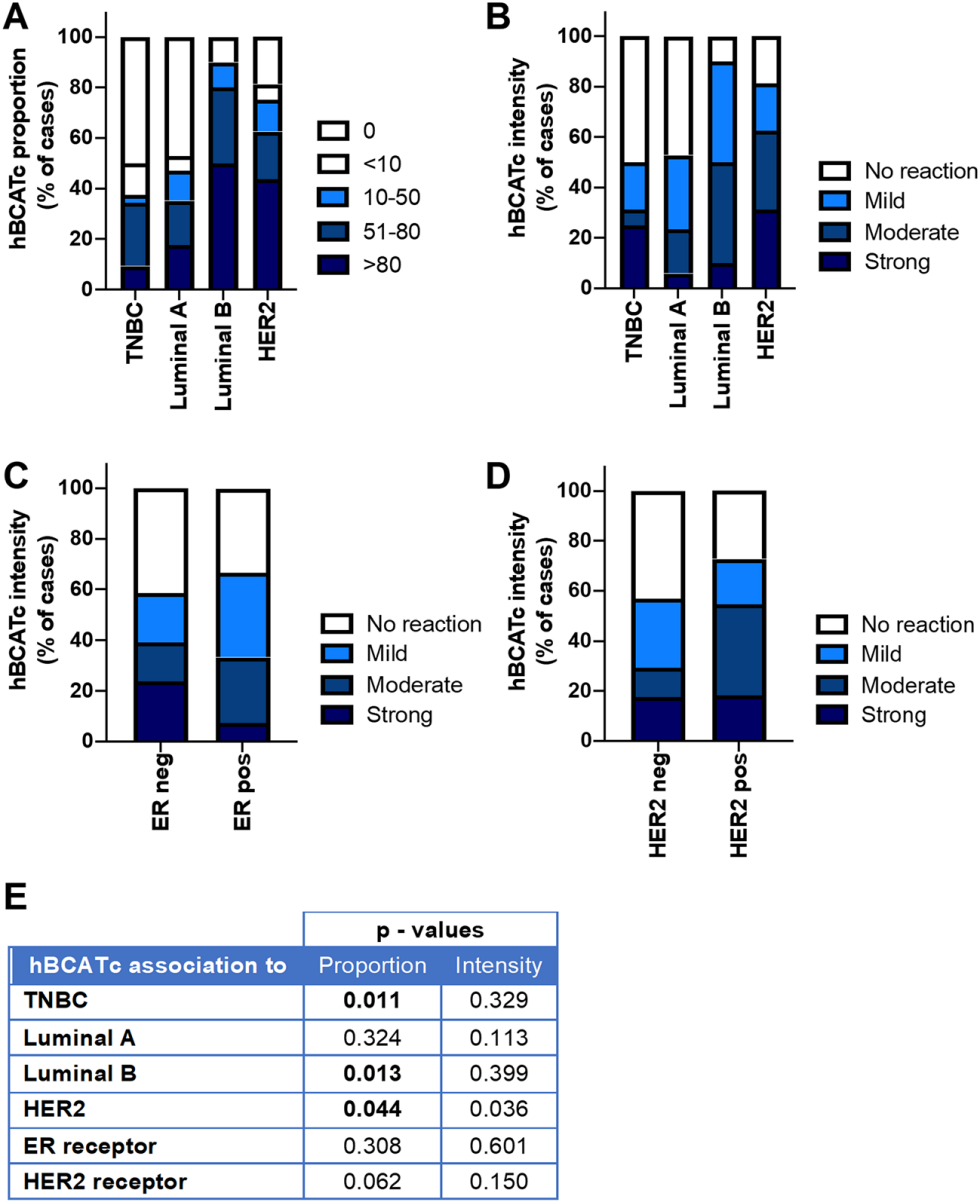
Proportion of cells expressing hBCATc and intensity of reaction was significantly associated with HER2 subtype. **a, b** Bar chart showing the percentage of cases of hBCATc (**a**) proportion and (**b**) intensity of the immunoreactivity for each of the subtypes of breast cancer (**c**) bar chart of percentage of hBCATc staining intensity between ER receptor negative and positive tumours (**d**) bar chart of percentage of hBCATc staining intensity between HER2 receptor negative and positive tumours (**e**) summary of the *p* values for the chi-square linear-by-linear test of association for hBCATc proportion and intensity association to TNBC, luminal A, luminal B and HER2 subtypes and to ER and HER2 receptor status

The expression of hBCATc was assessed in a total of 75 primary breast cancer cases using immunohistochemistry (Table 3). 62.7% of cases were positive for hBCATc expression. Differential expression of hBCATc was observed between the subtypes of breast cancer. HER2-subtype tumours showed significantly higher percentage of expression and staining intensity of hBCATc (*p* = 0.044 and *p* = 0.036) (Fig. 2a and b). Luminal B tumours also presented with a higher percentage of cells expressing hBCATc (*p* = 0.013) compared with luminal A and TNBC (Fig. 2a). There was no association of hBCATc intensity and ER status (*p* = 0.601) (Fig. 2c) whilst higher hBCATc intensity was present in HER2 receptor-positive tumours (*p* = 0.062) (Fig. 2d) suggesting an association of hBCATc with the expression of the HER2 receptor. Triple-negative breast cancer cases showed a significantly lower proportion of cells expressing hBCATc as shown in Fig. 2e compared with the other breast cancer subtypes (*p* = 0.011). Together, these findings show hBCATc is increased in HER2-amplified breast cancer tumours.

### hBCATm and IDH1 expression associated with luminal B breast cancer subtype and ER/PR receptor status

**Fig. 3:**
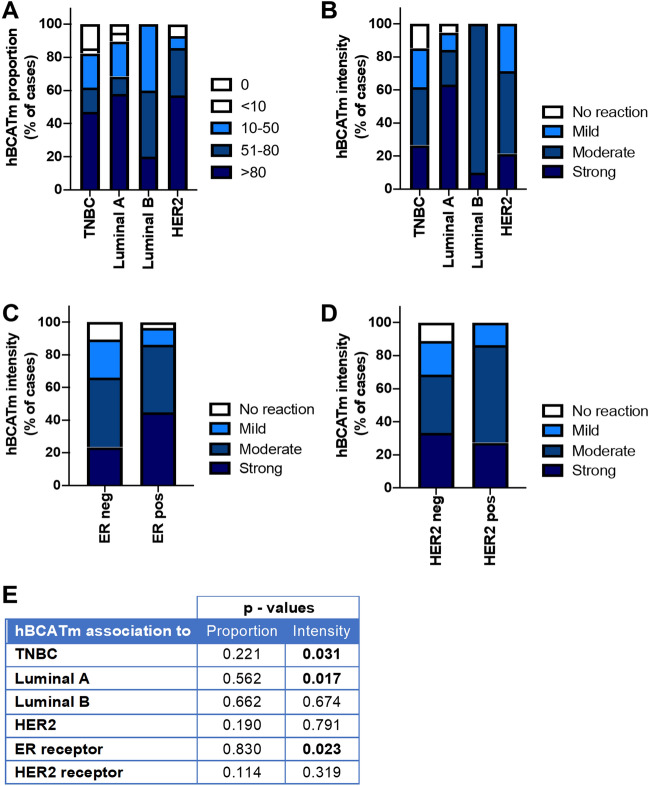
Proportion of cells expressing hBCATm was significantly associated with Luminal A subtype and ER receptor status. **a, b** Bar chart showing the percentage of cases of hBCATm (**a**) proportion and (**b**) intensity of the immunoreactivity for each of the subtypes of breast cancer (**c**) bar chart of percentage of hBCATm staining intensity between ER receptor negative and positive tumours (**d**) bar chart of percentage of hBCATm staining intensity between HER2 receptor negative and positive tumours (**e**) summary of the *p* values for the chi-square linear-by-linear test of association for hBCATm proportion and intensity associated to TNBC, luminal A, luminal B and HER2 subtypes and to ER and HER2 receptor status

The expression of hBCATm and IDH1 was positive in 88.3% and 90.8% of primary breast cancer cases, respectively (Table 3). Whilst hBCATc expression was found to be associated with HER2 subtype, expression of hBCATm and IDH1 metabolic proteins was found to be significantly increased in luminal A tumours compared with other breast cancer subtypes. Luminal A tumours expressed a higher level of hBCATm expression (Fig. 3b) with an association to ER status (*p* = 0.023) with no association to HER2 status (*p* = 0.319) (Fig. 3c and d). Although the proportion of cells expressing hBCATm did not significantly differ between tumour subtypes, luminal A tumour cells expressed a higher intensity of hBCATm expression (*p* = 0.017) (Fig. 3b) with a significant association to ER-positive status (*p* = 0.023) (Fig. 3c and e). Triple-negative breast cancer cases presented with significantly lower levels of hBCATm expression (*p* = 0.031).

**Fig. 4:**
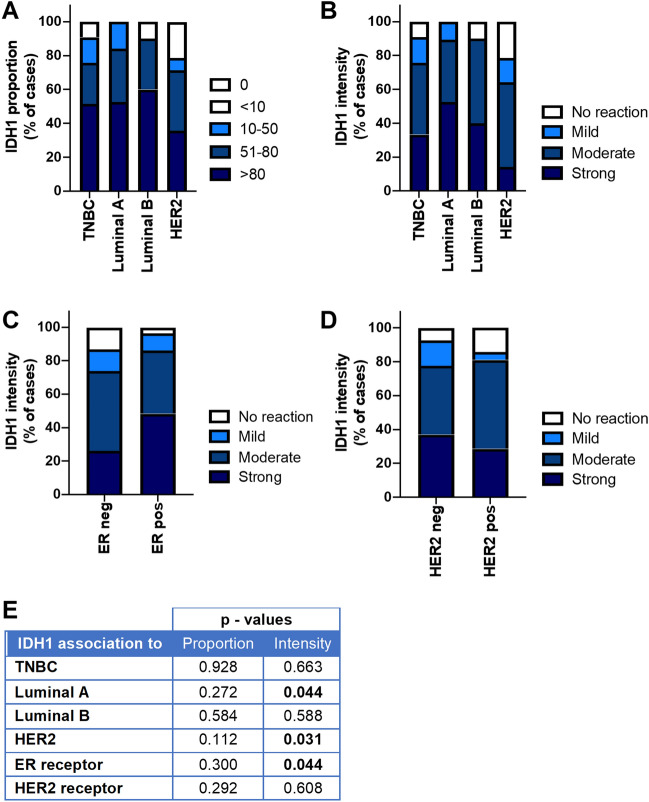
Proportion of cells expressing IDH1 intensity of reaction was significantly associated with Luminal A subtype. **a, b** Bar chart showing the percentage of cases of IDH1 (**a**) proportion and (**b**) intensity of the immunoreactivity for each of the subtypes of breast cancer (**c**) bar chart of percentage of IDH1 staining intensity between ER receptor negative and positive tumours (**d**) bar chart of percentage of IDH1 staining intensity between HER2 receptor negative and positive tumours (**e**) summary of the *p* values for the Chi-square linear-by-linear test of association for IDH1 proportion and intensity association to TNBC, luminal A, luminal B and HER2 subtypes and to ER and HER2 receptor status

**Fig. 5:**
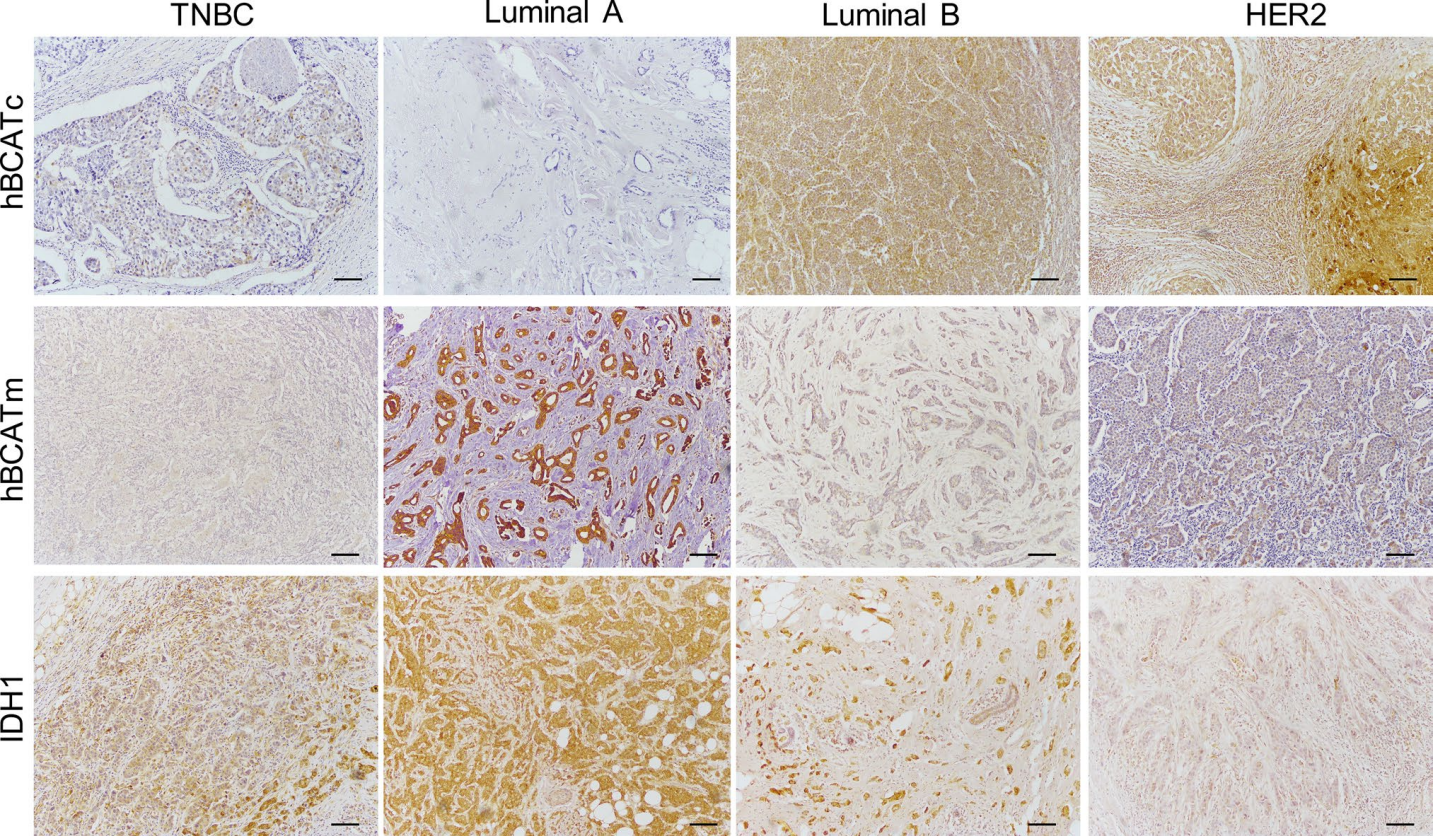
Representative images of the hBCAT and IDH1 proteins in different molecular breast cancer subtypes. hBCATc levels were significantly elevated in the HER-2 subtype and Luminal B subtypes. Luminal A subtype was characterised with increased levels of hBCATm and IDH1. × 10 objective (scale bars = 25 μm)

**Fig. 6:**
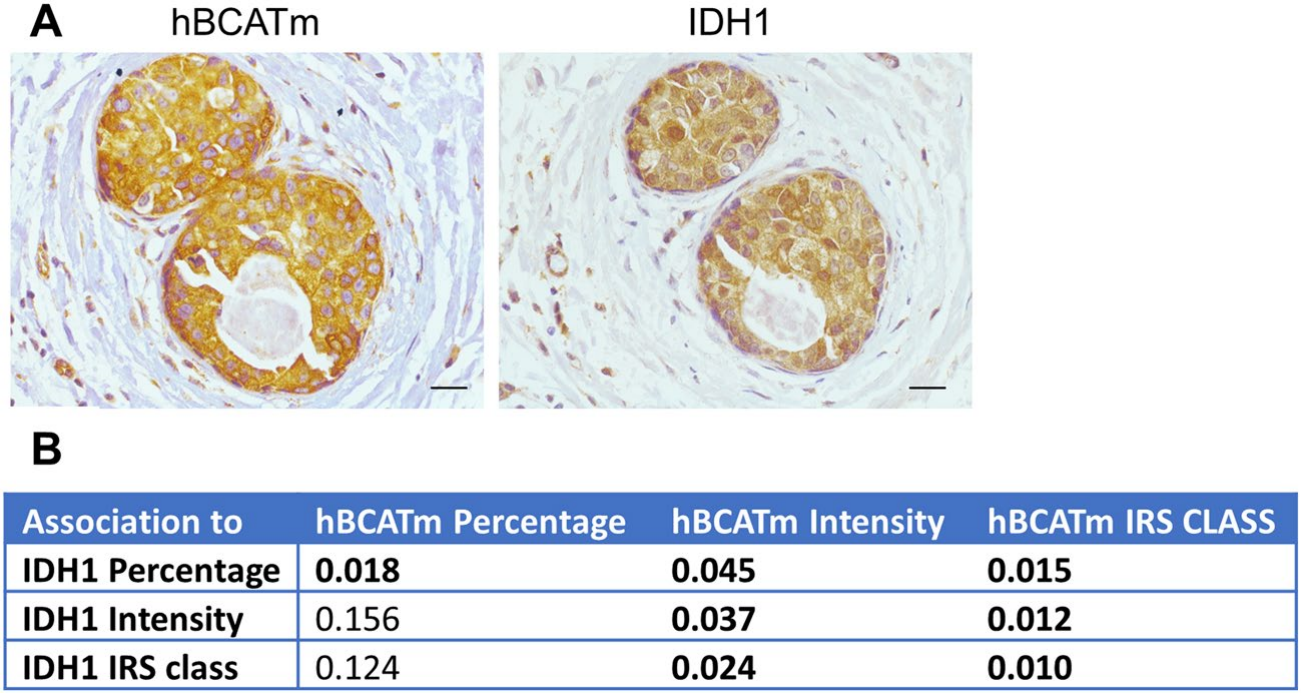
hBCATm and IDH1 co-expression was found to be significantly significant. **a** Co-expression of hBCATm and IDH1 was observed in the same cells. **b** Table showing the linear by linear p-values for hBCATm proportion, intensity and IRS class association with IDH1 expression. × 10 objective (scale bars = 250 μm)

Similar to hBCATm expression, IDH1 staining intensity was significantly higher in luminal A tumours (*p* = 0.044), whilst the proportion of cells expressing IDH1 did not differ between breast cancer subtypes as shown in Fig. 4a and b. IDH1 intensity associated with ER-positive status (*p* = 0.044) (Fig. 4c). In contrast, IDH1 intensity did not associate with HER2-positive status (*p* = 0.391) (Fig. 4d) and was lowest in the HER2 subtype (*p* = 0.031) (Fig. 4e). Hence, IDH1 expression association with luminal A subtype was confirmed to be associated with ER-positive expression rather than HER2-positive status. The differential expression of the hBCAT and IDH1 proteins between the breast cancer subtypes is represented in Fig. 5. Serial sections showed hBCATm and IDH1 to be expressed in the same cells indicating that their co-expression is important in the luminal A subtype, this association was found to be significant for both percentage and intensity of staining (*p* = 0.018 and *p* = 0.037) (Fig. 6).

### Association of metabolic enzymes to patient clinicopathological characteristics

**Fig. 7:**
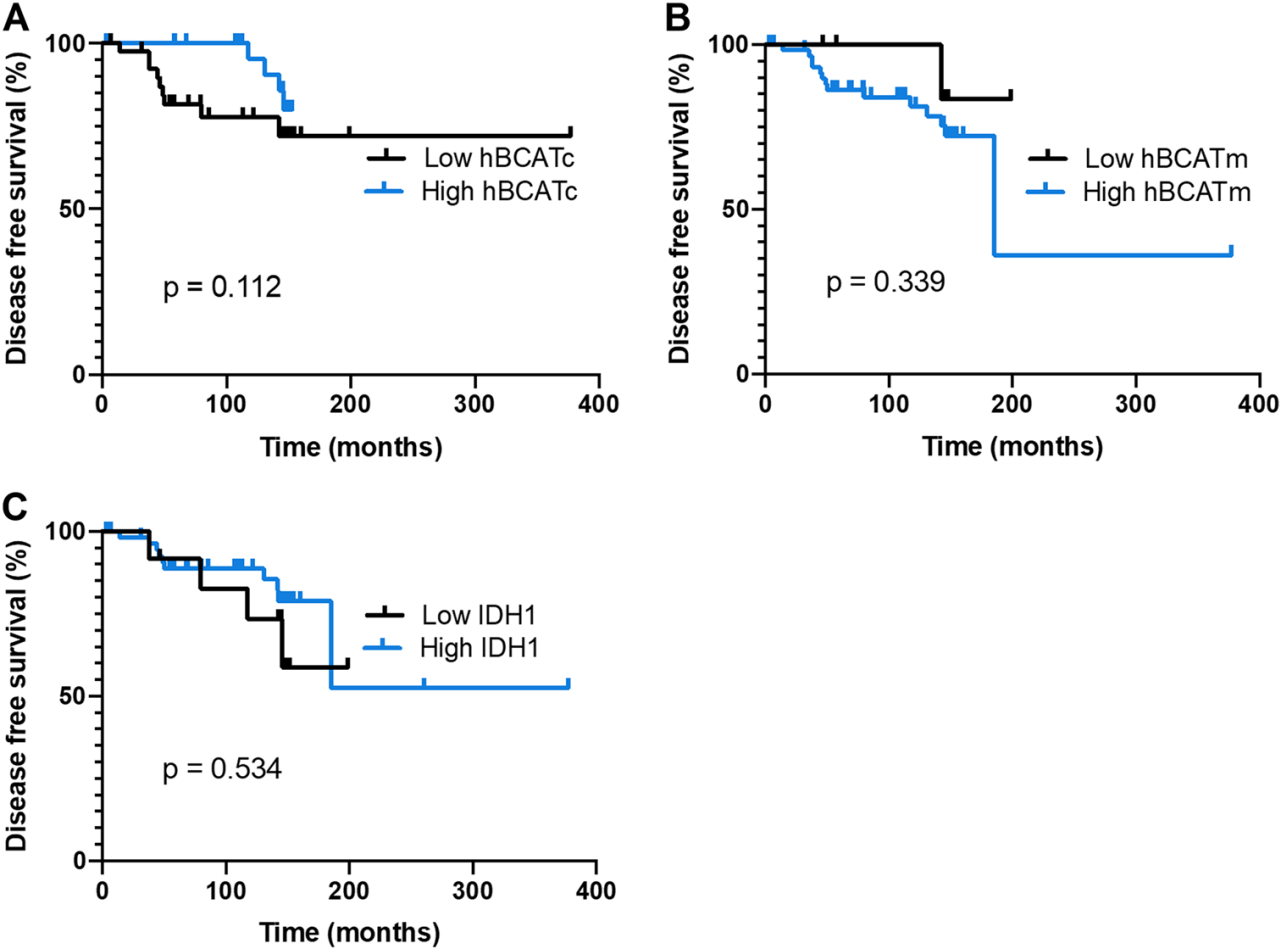
Disease-free survival for each of the metabolic proteins. Kaplan Meier analysis for **a** hBCATc **b** hBCATm **c** IDH1 low vs high total IRS class. Statistics were calculated from Log rank Mantel-Cox analysis

Statistical analysis was performed to assess the association between the expression of metabolic proteins; hBCATc, hBCATm and IDH1 to clinicopathological features (Tables 4, 5 and 6). Although expression of these metabolic proteins did not significantly associate with disease-free survival (Fig. 7), hBCATm was found to be significantly associated with smaller tumours (i = 0.029) with 91.8% of tumours ≤ 2 cm displaying moderate-to-strong IRS class compared with 73.4% of tumours > 2 cm. Similarly, IDH1 IRS class is significantly associated (*p* = 0.013) with small tumour size with 47.7% of larger tumours displaying strong IRS and only 20% of smaller tumours had a strong IRS class. IDH1 IRS class also associated with no lymph node invasion with 90.9% of cases having strong IRS. Lower histological grades were associated with strong IRS class of IDH1, 46.3% of cases compared with 22.9% of grade 3 tumours. Moreover, IDH1 strong IRS class also associated with lower TNM staging, in 52.9% of stage I tumours.

**Table 4:** Associations between hBCATc expression and clinicopathological characteristics

Parameters	Negative (IRS 0–1) (%)	Weak (IRS 2–3) (%)	Moderate (IRS 4–8) (%)	Strong (IRS 9–12) (%)	Total n	p value
Tumour type						0.291
Lobular	3 (42.9)	1 (14.3)	2 (28.6)	1 (14.3)	7	
Ductal	25 (40.3)	11 (17.7)	17 (27.4)	9 (14.5)	62	
Mixed/other	0 (0.0)	2 (33.3)	3 (50.0)	1 (16.7)	6	
Histological grade						0.852
I/II	16 (43.2)	2 (5.4)	15 (40.5)	4 (10.8)	37	
III	12 (31.6)	12 (31.6)	7 (18.4)	7 (18.4)	38	
Tumour stage						0.163
T1	19 (48.7)	3 (7.7)	13 (33.3)	4 (10.3)	39	
T2	9 (27.3)	10 (30.3)	7 (21.2)	7 (21.2)	33	
T3	0 (0.0)	1 (33.3)	2 (66.7)	0 (0.0)	3	
Nodal stage						0.989
N0	20 (40.8)	7 (14.3)	14 (28.6)	8 (16.3)	49	
N1	6 (33.3)	3 (16.7)	7 (38.9)	2 (11.1)	18	
N2	1 (20.0)	3 (60.0)	1 (20.0)	0 (0.0)	5	
N3	1 (33.3)	1 (33.3)	0 (0.0)	1 (33.3)	3	
Tumour stage (TNM)						0.254
I	16 (51.6)	2 (6.5)	11 (35.5)	2 (6.5)	31	
II	10 (29.4)	7 (20.6)	9 (26.5)	8 (23.5)	34	
III	2 (20.0)	5 (50.0)	2 (20.0)	1 (10.0)	10	
Tumour size (cm)						0.177
≤ 2	20 (46.5)	5 (11.6)	14 (32.6)	4 (9.3)	43	
> 2	8 (26.7)	8 (26.7)	8 (26.7)	6 (20.0)	30	
Lymph node invasion						0.461
No	19 (38.0)	7 (14.0)	15 (30.0)	9 (18.0)	50	
Yes	9 (36.0)	7 (28.0)	7 (28.0)	2 (8.0)	25	

Linear by linear association was used for statistical analysis

**Table 5:** Associations between hBCATm expression and clinicopathological characteristics

Parameters	Negative (IRS 0–1) (%)	Weak (IRS 2–3) (%)	Moderate (IRS 4–8) (%)	Strong (IRS 9–12) (%)	Total *n*	*p* value
Tumour type						0.757
Lobular	1 (11.1)	1 (11.1)	5 (55.6)	2 (22.2)	9	
Ductal	7 (11.3)	2 (3.2)	34 (54.8)	19 (30.6)	62	
Mixed/other	1 (16.7)	0 (0.0)	3 (50.0)	2 (33.3)	6	
Histological grade						0.078
I/II	2 (5.1)	1 (2.6)	23 (59.0)	13 (33.3)	39	
III	7 (18.4)	2 (5.3)	19 (50.0)	10 (26.3)	38	
Tumour stage						0.147
T1	4 (9.8)	0 (0.0)	21 (51.2)	16 (39.0)	41	
T2	5 (16.1)	2 (6.5)	18 (58.1)	6 (19.4)	31	
T3	0 (0.0)	1 (20.0)	3 (60.0)	1 (20.0)	5	
Nodal stage						0.348
N0	5 (10.2)	1 (2.0)	27 (55.1)	16 (32.7)	49	
N1	3 (16.7)	1 (5.6)	10 (55.6)	4 (22.2)	18	
N2	0 (0)	1 (14.3)	3 (42.9)	3 (42.9)	7	
N3	1 (33.0)	0 (0.0)	2 (66.7)	0 (0)	3	
Tumour stage (TNM)						0.254
I	3 (9.1)	0 (0.0)	17 (51.5)	13 (39.4)	33	
II	5 (15.6)	2 (6.3)	18 (56.3)	7 (21.9)	32	
III	51 (8.3)	1 (8.3)	7 (58.3)	3 (25.0)	12	
Tumour size (cm)						**0.029**
≤ 2	4 (8.9)	0 (0.0)	24 (53.3)	17 (37.8)	45	
> 2	5 (16.7)	3 (10.0)	17 (56.7)	5 (16.7)	30	
Lymph node invasion						0.937
No	7 (14.0)	1 (2.0)	26 (52.0)	16 (32.0)	50	
Yes	2 (7.4)	2 (7.4)	16 (59.3)	7 (25.9)	27	

Linear by linear association was used for statistical analysis

**Table 6:** Associations between of IDH1 expression and clinicopathological characteristics

Parameters	Negative (IRS 0–1) (%)	Weak (IRS 2–3) (%)	Moderate (IRS 4–8) (%)	Strong (IRS 9–12) (%)	Total n	p value
Tumour type						0.844
Lobular	0 (0.0)	1 (11.1)	4 (44.4)	4 (44.4)	9	
Ductal	7 (11.5)	4 (6.6)	29 (47.5)	21 (34.4)	61	
Mixed/other	0 (0.0)	0 (0.0)	4 (66.7)	2 (33.3)	6	
Histological grade						**0.046**
I/II	2 (4.9)	3 (7.3)	17 (41.5)	19 (46.3)	41	
III	5 (14.3)	2 (5.7)	20 (57.1)	8 (22.9)	35	
Tumour stage						0.079
T1	2 (4.9)	2 (4.9)	18 (43.9)	19 (46.3)	41	
T2	5 (16.1)	3 (9.7)	16 (51.6)	7 (22.6)	31	
T3	0 (0.0)	0 (0.0)	3 (75.0)	1 (25.0)	4	
Nodal stage						0.077
N0	2 (4.0)	4 (8.0)	21 (42.0)	23 (46.0)	50	
N1	4 (23.5)	0 (0.0)	11 (64.7)	2 (11.8)	17	
N2	1 (16.7)	1 (16.7)	3 (50.0)	1 (16.7)	6	
N3	0 (0.0)	0 (0.0)	2 (66.7)	1 (33.3)	3	
Tumour stage (TNM)						**0.022**
I	1 (2.9)	2 (5.9)	13 (38.2)	18 (52.9)	34	
II	5 (16.1)	2 (6.5)	17 (54.8)	7 (22.6)	31	
III	1 (9.1)	1 (9.1)	7 (63.6)	2 (18.2)	11	
Tumour size (cm)						**0.013**
≤ 2	2 (4.5)	2 (4.5)	19 (43.2)	21 (47.7)	44	
> 2	4 (13.3)	3 (10.0)	17 (56.7)	6 (20.0)	30	
Lymph node invasion						**0.018**
No	3 (5.9)	4 (7.8)	20 (39.2)	24 (47.1)	51	
Yes	4 (16.0)	1 (4.0)	17 (68.0)	3 (12.0)	25	

Significant p values are indicated in bold

Linear by linear association was used for statistical analysis

## Discussion

HER2 subtype, characterised by overexpression of its HER2 transmembrane receptor protein, is a higher-grade tumour with a more aggressive phenotype and worse prognosis relative to luminal A subtypes. Understanding the phenotypic profiling of breast cancer tumours is essential as it informs treatment regimens especially as different subtypes have distinct locoregional patterns [34]. Luminal A subtypes are at the lower risk of locoregional failure, and HER2-positive and TNBC at the higher risk. In this study, we show differential metabolic protein profiles for luminal A and HER2 tumour subtypes. A significant positive association between HER2 status and hBCATc was observed (Fig. 2) where, HER2 + breast cancer and TNBC subtypes demonstrated higher histopathological grading (*p* < 0.001) than the other subtypes (Table 1), indicating hBCATc expression is elevated in more aggressive tumours. Thewes et al*.* [23] have previously shown an upregulation of hBCATc in breast cancer tissue samples in ER-tumours, particularly TNBC and HER2 + subtype. In the cohort assessed, a significant association of hBCATc expression with HER2 + and luminal B breast cancer subtypes was demonstrated (Fig. 2). Whilst luminal B tumours may or may not overexpress HER2, hBCATc expression significantly associated with HER2 status which encompasses luminal B tumours with HER2 amplification, indicating HER2 receptor signalling in tumour may regulate hBCATc expression.

SKBR3 cells, which are a well-known breast cancer cell model for HER2 + subtype, have been characterised to lack hBCATc expression [23, 35]. Genome-wide proteomics of breast cancer cell lines, including SKBR3, reported hBCATc to correlate with higher levels of epidermal growth factor receptor (EGFR) expression, which is upregulated in TNBC, but not in HER2-positive breast cancer [35]. Conversely, Wang et al*.* [19] have demonstrated that hBCATc overexpression in the HER2-positive SKOV3 ovarian cancer cells contributes to increased cell proliferation, migration and invasion, thus facilitating tumour progression. Wilken et al*.* [36] found Herceptin, which is a commonly used HER2-targeted therapy, sensitised ovarian cancer (SKOV3) cells to EGFR-targeted therapy. Therefore, it is possible that elevated hBCATc expression observed in the HER2 breast cancer subtypes in vivo occurs in response to standard patient therapy. This suggests that the current breast cancer cell line models of HER2 + subtype do not reflect the pathophysiological process which has been observed in the human breast cancer tissue.

Despite the established link between *c-Myc* amplification and HER2 subtype, there are currently no studies directly attributing HER2 receptor amplification with hBCATc, which is up-regulated by c-Myc [37]. In contrast, much work has focused on re-programmed glutamine metabolism in cancer cells which promotes tumour energy generation, survival and growth [38]. Increased glutamine metabolic activity has been observed in HER2-positive breast cancer tissue compared with other subtypes of breast cancer, through upregulation of glutamine metabolic proteins including glutaminase 1 (GLS1) and glutamate dehydrogenase (GDH) [39, 40]. Furthermore, HER2 receptor induction in MCF-10A cells instigated upregulation of GLS1 and increased cell proliferation [41] establishing a direct role of HER2 in elevated glutamate synthesis. The transamination of BCAAs by hBCATc leads to the production of glutamate and the TCA intermediates acetyl-coA and succinyl-coA, which may be required to sustain tumour growth in this context. Glutamate offers multiple benefits to tumour growth as reviewed by [42] and elevated levels have been associated with breast cancer metastasis [43]. This indicates that hBCATc can function in conjunction with glutaminase to enhance glutamate flux to tumour cells and fuel cell growth through the TCA cycle. In addition to metabolite regulation, ligand binding to HER2 can activate the PI3K/Akt pathway, where a role for activated Akt in tumorigenesis is widely recognised. As discussed, we have evidenced that BCATc regulates cell proliferation and migration through activation of the PI3K/Akt pathway, whilst suppressing Ras/ERK activation, highlighting the plasticity of tumours to advance and adapt to changing environments [26], in particular in response to HER2 activation.

Although, immunohistochemical analysis of hBCATc expression in this cohort of breast cancer patients demonstrated a negative association with TNBC tumours (Fig. 2E), there was positive expression in 50% of the 32 TNBC cases assessed. Thewes et al*.* [23] have previously demonstrated an upregulation of hBCATc in a larger study in TNBC, whereby 75% of 109 TNBC tumours were positive for hBCATc expression. Moreover, expression of hBCATc has been demonstrated to be limited to the TNBC (MDA-MB-231) cells in a panel of breast cancer cell lines [23], further supporting a role for hBCATc in more aggressive subtypes. In gliomas, 2HG, which is a competitive inhibitor of multiple α-KG-dependent dioxygenases, produced by mutant-*IDH1*, has been found to inhibit *BCAT1* and *BCAT2* [31, 44]. Metabolomic analysis reported elevated levels of 2HG in the absence of *IDH-*mutations in MDA-MB-231 cells [32]. Elevated level of 2HG has been shown to promote H3K79 dimethylation [44]. Interestingly, *BCAT1* expression was upregulated by DOT1L-mediated histone dimethylation of H3K79 in MDA-MB-231 cells [45]. Thus, in breast cancer, epigenetic regulation mediated by 2HG and DOT1L may upregulate hBCATc in TNBC, which has been demonstrated to mediate proliferation and migration in breast cancer [23].

There is little evidence of *IDH1* mutations in breast cancer, with only one reported case discussed in the literature [32, 33]. Levels of 2HG are significantly increased in ER-tumours and breast cancer cell lines [32]. However, the only case of *IDH1* mutation was found to be reported in ER + breast cancer [33] indicating *IDH1* mutations to be a rare event in breast cancer. The IDH enzymes are responsible for the oxidative decarboxylation of isocitrate to produce α-ketoglutarate [46, 47]. IDH1 acts as a major source of cytosolic NADPH production required for multiple metabolic pathways, including glutathione production and fatty acid biosynthesis [48]. Low expression of IDH1 has been associated with poor prognosis in breast cancer, whilst high expression of IDH1 associated with better survival indicating that IDH1 acts as a tumour suppressor in breast cancer [49]. Correspondingly higher expression of IDH1 was found to be significantly associated with smaller tumour size, lower tumour stage and histological grade (Table 6) suggesting that IDH1 expression is indicative of a positive prognostic outcome.

For the first time, luminal A tumours were characterised by increased expression of hBCATm and IDH1 (Figs. 3 and 4). hBCATm was found to be significantly associated with IDH1 expression (Fig. 6), suggesting these two metabolic pathways are activated concomitantly. Indeed, sterol response element-binding protein 1 (SREBP1) has been demonstrated to regulate hBCATm, in pancreatic ductal adenocarcinoma [50] and IDH1, but not IDH2 expression, in breast cancer [51]. SREBP-1 is a transcription factor which activates genes involved in fatty acid synthesis and is reported to be upregulated in breast cancer [52]. Oestrogen stimulation in ER-positive (MCF7) cells has been shown to induce SREBP1 expression [53]. Knockdown of *BCAT2* in oestrogen receptor-positive (MCF7) cells was shown to result in reduced proliferation in contrast to normal-like MCF-10A breast cells and airway smooth muscle (ASM) cells where no effect on cell proliferation was observed [54] indicating tumour-selective *BCAT2*-mediated inhibition of cell growth.

Moreover, elevated oxidative stress in tumour cells can induce AMPK activation, which inhibits SREBP1 [55]. The thiol regions of the CXXC motif in hBCATc exhibit sensitivity to changes in the redox environment [56]. The hBCAT proteins contribute to glutathione (GSH) biosynthesis, by facilitating the uptake of cystine via the x_c_-cysteine transporter, which is coupled to the efflux of glutamate [31, 38]. GSH plays an important role in redox homeostasis and tumour cell survival by protecting cells from damage caused by the reactive oxygen species (ROS) generated during oxidative stress [57]. Moreover, expression levels of hBCAT have been demonstrated to regulate the expression of nuclear factor (erythroid-derived 2)-like-2 (Nrf2), which is the primary transcription factor responsible for the regulation of genes encoding oxidative stress‐related proteins [26]. Hence, increased expression of hBCATm in the luminal A subtype is suggested to reduce ROS levels, thereby providing a mechanistic positive feedback loop which enhances SREBP1-induced IDH1 and hBCATm upregulation. In gliomas, hBCATm has been identified as a negative prognostic marker in *IDH1*-WT gliomas [18], supporting the hypothesis for hBCATm to contribute to tumour progression in the absence of *IDH* mutation, which is a rare event in breast cancer [32, 33]. IDH1 is responsible for the oxidative carboxylation of isocitrate to maintain levels of α-ketoglutarate in the cytosol which is critical for the TCA cycle [58]. Conversely, hBCATm utilises α-ketoglutarate in the transamination of BCAAs in the mitochondria. Consequently, enhanced expression of IDH1 may replenish the α-ketoglutarate pool in cells with increased hBCATm, which can then be shuttled to the mitochondria via the membrane transporter SLC25A11 [58], to support the TCA cycle. Therefore, IDH1 and hBCATm metabolism can be proposed to work in conjunction to support tumour growth in luminal A tumours.

## Conclusion

In this study, hBCATc expression was found to be significantly associated with the more aggressive HER2 + and luminal B subtype whilst hBCATm and IDH1 to be significantly associated with luminal A subtype suggesting differential metabolic reliance between these two subtypes. For the first time a synergistic mechanistic expression of IDH1 and hBCATm in luminal A breast cancer has been described, which may be mediated by upregulation of the ER-activated SREBP1 transcription factor. Further work is needed to understand the mechanism by which HER2 may regulate hBCATc expression and the metabolic benefits hBCATc may offer to these tumours. Metabolic rewiring in breast cancer is suggested to be dependent on hormone and HER2 receptor expression. Thus, understanding these metabolic profiles will help provide subtype-specific therapeutic targets.

## Data Availability

The datasets during and/or analysed during the current study available from the corresponding author on reasonable request.

## Abbreviations

BCAA: Branched-chain amino acids
BCAT: Branched-chain aminotransferase
DAB: 3.3′-Diaminobenzidin
hBCATc: Human cytosolic branched-chain aminotransferase
hBCATm: Human mitochondrial branched-chain aminotransferase
BCKA: Branched-chain α-ketoacids
BCKDC: Branched-chain α-keto acid dehydrogenase enzyme complex
ER: Oestrogen
HER2: Human epidermal growth factor receptor-2
PR: Progesterone receptor
TNBC: Triple-negative breast cancer
IDH: Isocitrate dehydrogenase
2-HG: R-2-hydroxyglutarate
TBS: Tris-buffered saline
TCA: Tricarboxylic acid
